# A Comprehensive Review of Progressive Familial Intrahepatic Cholestasis (PFIC): Genetic Disorders of Hepatocanalicular Transporters

**DOI:** 10.14740/gr609e

**Published:** 2014-05-02

**Authors:** Syed Amer, Amtul Hajira

**Affiliations:** a Department of Internal Medicine, Mayo Clinic, Phoenix, AZ 85054, USA; b Department of Family Medicine, Carle Foundation Hospital, Urbana, IL 61801, USA

**Keywords:** Progressive familial intrahepatic Cholestasis, Cholestasis, Hepatocanalicular transporters

## Abstract

Progressive familial intrahepatic cholestasis or PFIC is a general term used to describe a group of genetic disorders involving the hepatocanalicular transporters. These diseases are characterized by persistent cholestasis, pruritus and jaundice. Type I PFIC is characterized by defect in the gene that codes for aminophospholipid translocase protein and maintains canalicular membrane stability. Types 2 and 3 are caused by defect in genes that code for bile acid transporter and a phospholipid translocase, respectively. This review summarizes the genetics, clinical features, diagnosis and treatment of the three types of PFIC.

## Introduction

Progressive familial intrahepatic cholestasis (PFIC) is a heterogeneous group of rare, genetic autosomal recessive disorders, resulting from defects in the mechanisms involved in bile formation with typical clinical, biochemical and histological features. These patients typically present with intrahepatic cholestasis in infancy or childhood. The course of the disease involves portal hypertension, liver failure, cirrhosis, hepatocellular carcinoma along with several extra hepatic manifestations. Three types of PFIC have been described: 1) PFIC type 1, caused by mutations in ATP8B1 gene (also called FIC1); 2) PFIC type 2, caused by mutations in ABCB11 gene (also called BSEP) and 3) PFIC type 3, caused by mutations in ABCB4 gene (also called MDR3). Each mutation is related to the hepatocellular transport system genes involved in bile formation [1, 2]. Cholestasis is a major clinical sign in all three types [3].

Table 1 summarizes the typical features of the three types of PFIC.

**Table 1:** Characteristic Features of the Three Types of Progressive Familial Intrahepatic Cholestasis

	PFIC 1	PFIC 2	PFIC 3
Inheritance	Autosomal recessive	Autosomal recessive	Autosomal recessive
Age of onset	Neonates	Neonates	1 month - 20 years
Gene	ATP8B1/F1C1	ABCB11/BSEP	ABCB4/MDR3
Chromosome	18q21-q22	2q24	7q21
Function of hereditary defect	Aminophospholipid translocase	Bile acid secretion	Phosphatidylcholine secretion
Cholestasis	Chronic	Chronic	Chronic
Pruritus	Severe	Severe	Moderate
Serum GGT	Normal	Normal	High
Serum ALT	Mildly elevated	> 5 × normal	> 5 × normal
Serum AFP	Normal	elevated	Normal
Serum primary bile acid (PBA) concentration	Very high	Very high	High
Lipoprotein X	Present	Present	Absent
Albumin	Low	Usually normal	Normal
Hepatocyte location	Canalicular membrane	Canalicular membrane	Canalicular membrane
Other sites of expression	CholangiocytesPancreasIntestines	None	None
Functional defect	ATP dependent aminophospholipid transport	ATP dependent bile acid transport in bile	ATP dependent phosphatidylcholine translocation in bile
Liver biopsy			
Histology	Minimal giant cell transformation, intracanalicular cholestasis, no ductal proliferation, minimal inflammation Late fibrosis	Giant cell transformation, intracanalicular cholestasis, no ductular proliferation, moderate inflammation, fibrosis	Giant cell transformation, intracanalicular cholestatis, ductular proliferation, moderate inflammation, marked fibrosis
Electron microscopy	Coarsely granular bile Loss of microvilli, swollen microvilli	Amorphous filamentous bile Loss of microvilli	Presence of cholesterol crystals Loss of microvilli
Immunohistochemistry	BSEP positive	BSEP negative	BSEP positive
	MDR3 positive	MDR3 positive	MDR3 negative
	GGT negative	GGT negative to weakly positive	GGT positive

BSEP: bile salt export protein; MDR3: multidrug resistance protein; GGTL: gamma glutamyl transpeptidase; ALT: alkaline tranferase; AFP: alpha fetoprotein.

## PFIC Type 1

### Genetics

PFIC1, an autosomal recessive disease is caused by the mutation in ATP8B1 gene, which encodes a P-type ATPase, located on human chromosome 18. It is found in several tissues such a small intestine, bladder, stomach, liver and pancreas. It is localized on the apical membrane of epithelial cells, including the canalicular membrane of hepatocytes [4, 5]. ATP8B1 is hypothesized to be an aminophospholipid flippase, translocating phospholipids such as phosphatidylserine from outer to the inner leaflet of plasma membrane [6]. Deficiency of ATP8B1 in the hepatocyte may result in loss of asymmetric distribution of the phospholipids in the canalicular membrane, thus reducing membrane stability and function of transmembrane transporters like ABCB11, impairing bile salt transport and decreasing hepatocyte resistance to bile salts, ultimately leading to reduced transport of bile salts in liver and gut resulting in cholestasis and watery diarrhea.

### Clinical features

In newborns afflicted with type 1 PFIC is characterized by recurrent episodes of intrahepatic cholestasis, intractable pruritus, diarrhea and failure to thrive in first several months of life. It is generally progressive leading to cirrhosis and eventually liver failure in the first decade of life. Other features include short stature, diarrhea, hepatosplenomegaly, malabsorption and occasionally hearing loss.

### Diagnosis

The diagnostic features include elevated serum bile salts, normal serum GGT, electron microscopy showing coarse granular bile and light microscopy showing bland cholestasis. Genetic testing is the only reliable way to distinguish between PFIC 1 and PFIC 2.

### Treatment

Pharmacological treatment includes ursodeoxycholic acid (UDCA) to promote biliary flow [7], but its effect on disease progression is uncertain. Rifampin (accelerates hepatic detoxification and excretion of bilirubin and bile salts) and cholestyramine (a hydrophilic, water insoluble anion exchange resin that binds bile salts, preventing their reabsorption in enterohepatic circulation) ease pruritus. These patients may also need supplements of fat soluble vitamins.

Partial biliary diversion may be beneficial in some patients, significantly decreasing their symptoms [8]. It may even delay or interrupt hepatocyte injury if performed early in the course of the disease. Liver transplantation is the most effective form of treatment especially in those with progressive disease or established cirrhosis, usually within the first decade of life.

## PFIC Type 2

### Genetics

PFIC 2, an autosomal recessive disease is caused by mutation in ABCB11 gene on chromosome 2q24, which encodes the canalicular bile salt export protein (BSEP), a liver specific adenosine triphosphate (ATP)-binding cassette transporter [9, 10]. BSEP is a major exporter of primary bile acids against extreme concentration gradients. There are many mutations that have been identified, but the most common mutations are the missense mutations E297G and D482G. Defective BSEP can lead to impaired bile salt secretion, accumulation of bile salts in hepatocytes and subsequent hepatocellular injury, apoptosis or necrosis. Missense mutations cause less severe disease, whereas the risk of hepatobiliary malignancy is increased with trunacating mutations.

### Clinical features

Patients usually present with cholestatic jaundice in neonatal period. Pruritus is usually the dominant feature. Clinical features and disease progression may be more severe in PFIC 2 than in PFIC 1. Other features may include growth failure, deficiency of fat soluble vitamins. These patients are at considerable risk of hepatobiliary malignancy, hepatocellular carcinoma or cholangiocarcinoma , thus close surveillance is important in these patients [11].

### Diagnosis

Diagnostic features include elevated bile salts, normal serum GGT, electron microscopy showing amorphous or filamentous bile. Light microscopy shows nonspecific giant cell hepatitis. Immunohistochemical staining for canalicular BSEP is negative in majority of patients.

### Treatment

Patients with PFIC 2 have poor response to ursodeoxycholic acid. Partial external biliary diversion (PBED) (it interrupts the enterohepatic circulation by partially diverting bile from the gallbladder through a loop of jejunum connecting the gallbladder to abdominal skin) may be useful and if performed early enough may delay or interrupt hepatic injury. Histological changes may show some improvement.

Liver transplantation is usually successful in this group of patients although recurrence after transplantation has been reported [12]. This can be resolved with aggressive immunosuppressive therapy.

## PFIC Type 3

### Genetics

PFIC 3, an autosomal recessive disease is caused by mutations in ABCB4, which encodes the multidrug resistance-3 gene (MDR3), an ATP-binding cassette transporter. It is located on chromosome 7q21. It acts as a phospholipid translocator involved in biliary phospholipid (phosphatiylcholine) excretion. It is mainly expressed in the hepatocyte canalicular membrane. ABCB4 defects cause impaired excretion of phophatidycholine, which is an important component in formation of mixed micelles, which protect the membrane of cells facing biliary tree from the detergent activity of bile salts. Functional deficiency of ABCB4 causes the detergent action of bile salts to solubilize the cell membrane of the biliary epithelium inducing inflammation and cell death.

Several different types of mutation have been identified and those with missense mutation have less severe phenotype, with later onset of disease, slower progression and better response to treatment than those with trunacating mutations.

Heterozygous mutations in ABCB4 gene can cause conditions such as intrahepatic cholestasis of pregnancy, cholesterol gall stone formation, drug induced cholestasis and adult biliary cirrhosis [13].

### Clinical features

Patients with ABCB4 deficiency usually have jaundice, pale stools, hepatosplenomegaly or pruritus. Age at onset varies from infancy to adulthood. Adolescent and young adult patients are more likely to have cirrhotic symptoms owing to portal hypertension. No association with malignancy has been described in PFIC 3 patients [14].

### Diagnosis

The diagnostic features include elevated serum bile salts, elevated serum GGT, electron microscopy showing presence of cholesterol crystals and loss of microvilli, light microscopy showing ductular proliferation and inflammatory infiltrate in early stages, portal and peri-portal fibrosis, cirrhosis and cholestasis which are seen in the later stages.

### Treatment

UDCA is useful in almost half of the patients. It is more likely to work in those who have an MDR3 missense mutation as they have some residual protein activity. Those who do not respond to UDCA treatment are likely to have a truncated protein. In these patients, liver transplantation is the primary treatment.

Table 2 lists the differential diagnosis of the three types of PFIC that an astute clinical should be mindful of.

**Table 2:** Differential Diagnosis of PFIC

A) Disorders of bile acid synthesis biosynthesis and conjugation
i) 3-oxo-4-steroid 5-reductase deficiency
ii) 3 -hydroxy-5-C27-steroid dehydrogenase/isomerase deficiency
iii) Oxysterol 7-hydroxylase deficiency
iv) Bile acid-CoA: amino-acid N-acyltransferase deficiency (familial hypercholanemia)
B) Disorders of embryogenesis
i) Alagille syndrome (jagged 1 defect, syndromic bile duct paucity)
ii) Ductal plate malformation (autosomal recessive polycystic kidney disease, autosomal dominant polycystic liver disease, Caroli’s disease)
C) Trafficking and canalicular targeting defects
i) Arthrogryposis, renal dysfunction, cholestasis
D) Tight, junction defects
i) Neonatal ichthyosis sclerosing cholangitis
ii) Familial Amish hypercholanemia
E) Miscellaneous
i) Aagenaes syndrome ( hereditary cholestasis with lymphedema)
ii) North American Indian Childhood Cirrhosis

## Conclusion

The review aims to summarize the genetics, clinical features, diagnostic and therapeutic strategies for the three types of PFIC. Further research is needed to elucidate the disease mechanism in these disorders. There is also an urgent need to develop mutation specific pharmacological therapies.
